# Ten-eleven translocation 1 mediating DNA demethylation regulates the proliferation of chicken primordial germ cells through the activation of Wnt4/β-catenin signaling pathway

**DOI:** 10.5713/ab.23.0310

**Published:** 2024-01-20

**Authors:** Yinglin Lu, Ming Li, Heng Cao, Jing Zhou, Fan Li, Debing Yu, Minli Yu

**Affiliations:** 1 Department of Animal Genetics, Breeding and Reproduction, College of Animal Science and Technology, Nanjing Agricultural University, Nanjing, Jiangsu 210095, China

**Keywords:** β-Catenin, Demethylation, Primordial Germ Cells (PGCs), Proliferation, Ten-eleven Translocation 1 (*Tet1*)

## Abstract

**Objective:**

The objective of this study was to investigate the regulation relationship of Ten-eleven translocation 1 (*Tet1*) in DNA demethylation and the proliferation of primordial germ cells (PGCs) in chickens.

**Methods:**

siRNA targeting *Tet1* was used to transiently knockdown the expression of *Tet1* in chicken PGCs, and the genomic DNA methylation status was measured. The proliferation of chicken PGCs was detected by flow cytometry analysis and cell counting kit-8 assay when activation or inhibition of Wnt4/β-catenin signaling pathway. And the level of DNA methylation and hisotne methylation was also tested.

**Results:**

Results revealed that knockdown of *Tet1* inhibited the proliferation of chicken PGCs and downregulated the mRNA expression of *Cyclin D1* and cyclin-dependent kinase 6 (*CDK6*), as well as pluripotency-associated genes (*Nanog*, *PouV*, and *Sox2*). Flow cytometry analysis confirmed that the population of PGCs in *Tet1* knockdown group displayed a significant decrease in the proportion of S and G2 phase cells, which meant that there were less PGCs entered the mitosis process than that of control. Furthermore, *Tet1* knockdown delayed the entrance to G1/S phase and this inhibition was rescued by treated with BIO. Consistent with these findings, Wnt/β-catenin signaling was inactivated in *Tet1* knockdown PGCs, leading to aberrant proliferation. Further analysis showed that the methylation of the whole genome increased significantly after *Tet1* downregulation, while hydroxymethylation obviously declined. Meanwhile, the level of H3K27me3 was upregulated and H3K9me2 was downregulated in *Tet1* knockdown PGCs, which was achieved by regulating Wnt/β-catenin signaling pathway.

**Conclusion:**

These results suggested that the self-renewal of chicken PGCs and the maintenance of their characteristics were regulated by *Tet1* mediating DNA demethylation through the activation of Wnt4/β-catenin signaling pathway.

## INTRODUCTION

In the germ lineage, changes in DNA methylation/demethylation occur during the formation of primordial germ cells (PGCs) to reset the epigenome of future gametes [1,2]. The TET family members (TET1, 2, and 3) have the capacity to oxidize 5-methylcytosine (5mC) to 5-hydroxymethylcytosine (5hmC), which exerts important roles in epigenetic reprogramming in early embryos and PGCs [3,4]. In our previous study, knockdown of Ten-eleven translocation 1 (*Tet1*) showed impaired survival and proliferation in PGCs, as well as increased 5mC level and reduced 5hmC level [5]. However, the detailed mechanism of this process has not yet been well characterized in chicken PGCs. The present study focused on the molecular regulation of *Tet1* mediating demethylation in proliferation and pluripotency in chicken PGCs.

DNA methylation is an important epigenetic modification, which plays a vital role in gene regulation and transposable element silencing [6]. How DNA methylation is reversed is a central question in epigenetic reprogramming [7]. TET1 contains a core catalytic domain and a chromatin-associated CXXC domain to bind CpG sequences which play essential roles in PGC development [8]. The expression of *Tet1* is upregulated in reprogramming germ cells, and the locus-specific DNA demethylation in genes related to meiosis was affected by targeted deletion of *Tet1* in mice [8,9]. Increasing evidence indicates that TET1 is involved in the epigenetic regulation of proliferation and differentiation in various cells such as embryonic stem cells (ESCs), adult neural progenitor cells, muscle progenitor cells, and cancer cells [10,11]. Mouse PGCLCs effectively recapitulated the genome-wide DNA demethylation occurring in the intragonadal PGCs [12].

Wnt/β-catenin signaling pathway plays a crucial role in PGC development, including specification, migration, and proliferation in PGCs [13]. A recent study demonstrated that loss of TET1 induced aberrant DNA methylation and exhibited suppressor function through regulating Wnt signaling pathway in colon cancer cells [14]. JW74, a β-catenin inhibitor, was diminished through altering histone modifications [15]. JW74 stabilizes AXIN, a component of the β-catenin degradation complex, eventually causing β-catenin degradation [16], whereas BIO led to target gene expression by stabilizing β-catenin from the degradation complex [17]. However, to which degree the Wnt/β-catenin signaling pathway is activated by *Tet1* and how it affects the development of PGCs remains unclear.

DNA methylation patterns and histone marks are globally remodeled in PGCs when they migrate to the genital ridges [18,19]. Factors involved in deposition of epigenetic marks regulate the properties related to self-renewal and pluripotency in PGCs. Trimethylation of histone H3 on lysine 27 (H3K27me3) by polycomb group proteins is involved in several epigenome-remodeling steps [20]. The interplay between H3K27me/PcG and DNA methylation also play important work during PGC expansion and migration [21]. A previous study showed that PGCs undergo genome demethylation via the 5hmC intermediate before an increase in the level of H3K27me3 [21]. There are few available data on the epigenome focused on DNA methylation in chicken germ line [5,18].

In mouse PGCs, a global gain in H3K27me3, concomitant with a loss of H3K9me2, occurs after specification and before the entry of these migratory cells into the gonads, where epigenetic reprogramming takes place [21]. In chicken PGCs, the H3K27me3 global level was greatly reduced, whereas the H3K9me3 level was elevated [20]. From these studies, a wide range of mechanisms for achieving demethylation have been proposed that may operate *in vivo* in the germline [22]. However, mechanisms coordinating these processes remain unclear.

To gain insights into the mechanism by which *Tet1* contributes to the proliferation of PGCs, the early molecular events that underlie demethylation in PGCs were investigated. The results demonstrated that *Tet1* was required for 5hmC accumulation, which play essential roles in the regulation of proliferation and pluripotency in chicken PGCs by mediating the Wnt4/β-catenin signaling pathway. The present study revealed the key function of *Tet1* in regulating the demethylation of PGCs genome and provided a theoretical foundation of the regulation of germ cell development by using PGCs culture model.

## MATERIALS AND METHODS

### Experimental animals

All procedures were implemented according to the Local Experimental Animal Care Committee and approved by the ethics committee of Nanjing Agricultural University (Nanjing, China; SYXK-2019-00085).

### Culture and treatment of chicken primordial germ cells

Fertilized Hyline chicken eggs were incubated at 38.5°C and 60% humidity in an egg incubator (BSS160; Grumbach, Mücke, Germany) according to the standard operating protocols. PGCs were isolated from embryonic genital ridges at E4.5 using our standard protocol [23]. The culture medium was α-MEM (1257107; Gibco, Thermo Fisher Scientifc, Waltham, MA, USA) supplemented with 15% knockout serum replacement (KSR, 10828-028; Invitrogen, Carlsbad, CA, USA), 10 ng/mL leukemia inhibitory factor, 10 ng/mL human bFGF (060-04543; Wako Pure Chemicals, Osaka, Japan), and 2% chicken embryo extract. Chicken PGCs were cultured in an incubator maintained at 37°C with an atmosphere of 5% CO2 and 60% to 70% relative humidity. Anti-stage-specific embryonic antigen (SSEA-1) antibody was used to identify and sort PGCs.

To test the role of Wnt4/β-catenin in *Tet1*-mediated PGC proliferation, specific activator 6-bromoindirubin-3′-oxime (BIO, 0, 0.5, 1.0, or 1.5 μM) and inhibitor JW74 (0, 5, 10, or 15 μM) (Sigma-Aldrich Chemical Company, St.Louis, MO, USA) were used to detect their effects. BIO and JW74 were dissolved in dimethyl sulfoxide (DMSO), and the control received the vehicle only with a final DMSO concentration of <0.1% that had no significant effect on cell proliferation and survival.

### Knockdown of *Tet1* in PGCs using siRNA

For RNA interference assays, *Tet1*-specific siRNAs were designed according to our previous study [5]. Cells were plated in 12-well plates at 5×10 cells per well and transfected with 50 nM indicated siRNAs plus siRNA mate (GenePharma, Shanghai, China) according to the manufacturer’s protocol. PGCs were sorted with fluorescence-activated cell sorting (FACS) after transfection and replated for 48 h before being harvested and used for characterizations and further measurement.

### Isolation of RNA and quantitative real time polymerase chain reaction

Total RNA was extracted from each sample with Trizol reagent (Invitrogen, USA). Quality and purity were determined by the Nanodrop-2000 spectrophotometer (Thermo Scientific, Wilmington, DE, USA), and the integrity was detected by 1.5% agarose gel-electrophoresis. RNA (500 ng) was reverse transcribed to cDNA after DNase treatment by using 5× All-In-One RT MasterMix (Applied Biological Materials Inc., Richmond, BC, Canada) according to the manufacturer’s instructions. Quantitative real time polymerase chain reaction (qRT-PCR) was performed by using SYBR Premix Ex TaqTM kit (Takara, Dalian, China) on an ABI7500 Fast thermal cycler (Applied Bio-systems Biosystems, Foster City, CA, USA) by following the manufacturer’s instructions. The final PCR reactions contained 10 μL 2×SYBR Premix Ex Taq II (Tli RNaseH Plus), 0.8 μL of forwards and reverse primer (10 mM), 2 μL first-strand cDNA and 6.4 μL H_2_O. Cycling parameters were 95°C for 30 s, followed by 40 cycles at 95°C for 15 s and 60°C for 30 s. Primer specificity was determined by melt curve analysis. Both no-reverse-transcription and no-template controls were used to confirm a lack of genomic DNA contamination and primer dimerization. All samples were normalized with the *β-actin* using the comparative cycle threshold method (2^–ΔΔCt^). Primer sequences are listed in Table 1. Differences with p<0.05 were considered as significantly different.

### Immunofluorescence staining

Chicken PGCs were grown on gelatinised glass coverslips, washed with phosphate-buffered saline (PBS), and fixed in 4% paraformaldehyde for 20 min at room temperature (RT). Cells were permeabilized with 0.55 Triton X-100 in PBS for 30 min at RT, washed with PBS and saturated with 2% bovine serum albumin (BSA) in PBS for 1 h. Then PGCs were incubated with the primary antibody raised against anti-SSEA-1 antibody (1:1,000; Developmental Studies Hybridoma Bank, Iowa, IA, USA), 5mC and 5hmC (1:1,000; Abcam, Cambridge, UK) overnight at 4°C in the blocking solution. After three washes with 0.1% Tween-20 in PBS, PGCs were incubated in Alexa-IgG-568 antibodies, fluorescein isothiocyanate-conjugated goat anti-mouse immunoglobulin M (IgM), or goat anti-mouse IgG (1:1,000; KPL Inc., Gaithersburg, MD, USA) as the secondary antibody in the blocking solution for 1 h. The nuclei were counterstained with 4′, 6-diamidino-2-phenylindole (DAPI, 300 nm; Sigma Aldrich, St. Louis, MO, USA). After a brief wash in PBS, samples were mounted with SlowFade Gold antifade reagent (Invitrogen, USA). For the detection of 5mC and 5hmC, nuclear DNA was denatured with 2 N HCl for 30 min and then neutralized with 100 mM Tris-HCl (pH 8) for 10 min. Finally, the images were visualized using laser-scanning confocal microscopy (Zeiss LSM 700; Carl Zeiss AG, Oberkochen, Germany).

### Western blot analysis

PGCs were sorted by FACS after treatment and lysed in radioimmunoprecipitation assay lysis buffer supplemented with protease inhibitors (Complete; Roche, Shanghai, China) and 0.2 nM phenylmethyl sulfonyl fluoride. Protein (20 ng) was denatured and subjected to 10% sodium dodecyl sulfate/polyacrylamide gel electrophoresis. After transfer to polyvinylidene fluoride membrane, the membrane was incubated in 5% dry skim milk in tris buffered saline (0.1% Tween-20) at RT for 1 h. The primary antibodies were rabbit anti-phospho β-catenin (1:1,000; Abcam, UK), rabbit anti-WNT4 (1:1,000; Abcam, UK) and β-actin (1:5,000; Novus Biologicals, Littleton, CO, USA) at 4°C overnight. After 1 h incubation with anti-mouse IgG or anti-rabbit IgG peroxidase-conjugated second antibody (1:5,000; Santa Cruz, Santa Cruz, CA, USA), blots were developed by enhanced chemiluminescence (Thermo Fisher Scientific, Waltham, MA, USA) and exposed to a ChemiDoc-XRS System (Bio-Rad, Hercules, CA, USA) to detect chemiluminescence.

### Cell proliferation assay

The proliferation of PGCs was determined using the cell counting kit-8 (CCK8) assay (Dojindo, Kyushu, Japan) according to the manufacturer’s protocol. Briefly, 1×10^4^ cells were seeded into 96-well plate and transfected with siRNAs. After transfection for 48 h, fresh culture medium with 10 mL CCK8 solution was added and incubated for another 2 h at 37°C. The optical density (OD) of each well was measured at 450 nm using an automated microplate reader (Thermo Scientific Multiskan GO microplate spectrophotomete).

### Flow cytometry analysis

For the cell cycle analysis, PGCs were first dissociated into single cells and fixed in 70% ethanol at −20°C for 30 min after washing with ice-cold PBS. The cells were incubated with staining solution (50 μg/mL propidium iodide, 0.1% Triton X-100 and 100 mg/mL RNase A in PBS) for 15 min at 4°C. The stained cells were analyzed by flow cytometry (BD FACSCELLULAR, Franklin Lakes, NJ, USA). Data was analyzed using FACSDiva and Modfit LT cell-cycle analysis software (Verity Software House, Topsham, ME, USA).

### Genomic DNA extraction and dot blot analysis

To detect 5mC and 5hmC levels, genomic DNA was extracted by phenol-chloroform-isoamyl alcohol, and the concentration of samples was serially diluted twofold. Dot blot analysis was performed as described previously [5]. Subsequently, genomic DNA was spotted on a positively charged nylon membrane (Roth, Karlsruhe, Germany), air dried for 15 min and cross-linked using the UV light (20 s, 1,200 J/cm^2^). Then membranes were blocked in 5% non-fat milk in PBS/0.1% Tween-20 for 1 h at RT before incubation incubated with anti-5mC (1:5,000; Abcam, UK) antibody or anti-5hmC (1:5,000; Active Motif, Carlsbad, CA, USA) overnight at 4°C followed by washing in PBS for 3 times. The membrane was then incubated with HRP-labeled anti-mouse/rabbit secondary antibody (1:5,000; Santa Cruz, USA) and signal was developed using an enhanced chemiluminescence reagent (Bio-Rad, USA). The intensity of signal was measured using Image J software and calibrated against the linear range of the standard curves to estimate the quantities of 5mC or 5hmC in each sample.

### Statistical analysis

Statistical analysis was performed using SAS version 9.4 software (SAS Institute, Cary, NC, USA). When a significant main effect was detected by analysis of variance using SAS, the least-squares analysis was used to compare the different treatments. Differences between control and treatment groups were deemed to be significant when p<0.05.

## RESULTS

### *Tet1* knockdown reduced the pluripotency of PGCs

**Figure 1:**
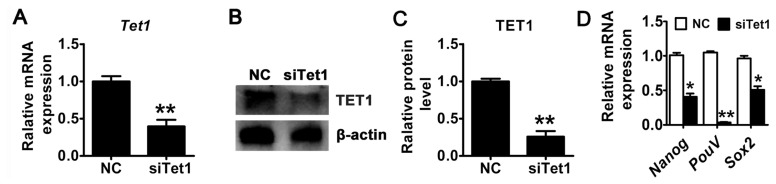
Knockdown of *Tet1* in PGCs. The mRNA (A) and protein (B)-(C) expression level of *Tet1* was measured. (D) The expression of pluripotency-related genes *PouV*, *Nanog*, and *Sox2* was detected by qRT-PCR. All the results represent the mean±standard deviation of three independent experiments (n = 3). *Tet1*, translocation 1; PGCs, pimordial germ cells; qRT-PCR, quantitative real time polymerase chain reaction; *PouV*, POU domain class 5; *Nanong*, Nanog homeobox; *Sox2*, Sex-determining region Y-box2. * p<0.05; ** p<0.01.

To investigate the role of *Tet1* in the pluripotency of PGCs, siRNA targeting *Tet1* was used to transiently knockdown of *Tet1* in chicken PGCs. Both mRNA and protein expression of Tet1 in PGCs was examined after *Tet1* siRNA transfection for 48 h. The results demonstrated that the mRNA expression level of *Tet1* decreased approximately by 50% in *Tet1* knockdown group compared with control (Figure 1A) (p< 0.01). In addition, the reduction of TET1 protein was observed by western blotting analysis (Figure 1B). Introduction of siRNA resulted in a 3-fold reduction in the level of TET1 (Figure 1C) (p<0.01). Furthermore, the expression of *Nanog*, *PouV*, and *Sox2* was declined significantly after the down-regulation of *Tet1* (Figure 1D) (p<0.05).

### *Tet1* knockdown increased the genomic DNA methylation

**Figure 2:**
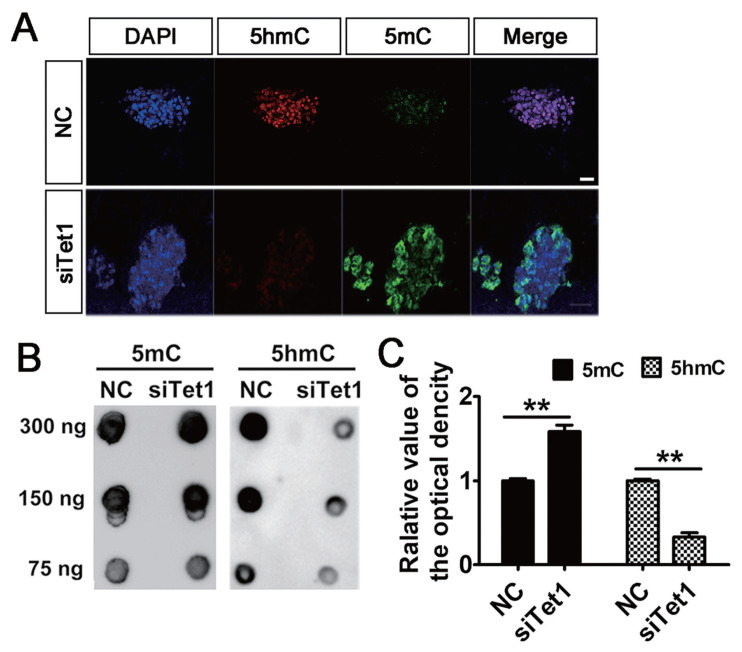
Effects of *Tet1* knockdown on DNA methylation in PGCs. (A) *Tet1* knockdown led to the increase of 5mC (green) and reduction of 5hmC (red) levels in PGCs by immunofluorescence. Scale bar, 20 μm. (B)-(C) The level of 5mC and 5hmC in si*Tet1* PGCs was confirmed by DNA dot blot. All the results represent the mean±standard deviation of three independent experiments (n = 3). *Tet1*, translocation 1; PGCs, primordial germ cells; 5mC, 5-methylcytosine; 5hmC, 5-hydroxymethylcytosine. ** p<0.01.

To address the involvement of *Tet1* in the level of genomic DNA methylation, the status of 5mC and 5hmC in PGCs was measured by immunostaining and DNA dot blot. Results showed that knockdown of *Tet1* led to a general increase in level of 5mC and the level of 5hmC diminished in PGCs (Figure 2A). A similar distribution of 5mC and 5hmC in PGCs was confirmed by DNA dot blot analysis. Expectedly, it was found that *Tet1* knockdown induced significant increase in 5mC level in si*Tet1* group compared with control (Figure 2B–2C) (p<0.01). By contrast, knockdown of *Tet1* induced significant decrease 5hmC level in si*Tet1* group (Figure 2B–2C) (p<0.01). This suggested that the potent capacity of PGCs to induce demethylation was restricted in *Tet1* knockdown group.

### *Tet1* exerted its effect via Wnt4/β-catenin signaling pathway

**Figure 3:**
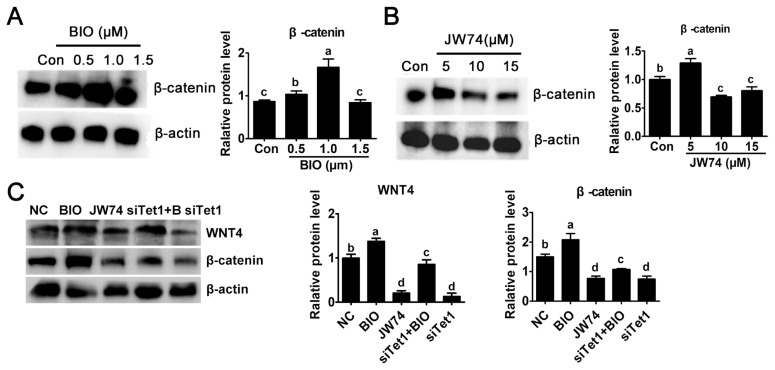
Effects of *Tet1* knockdown on Wnt4 and β-catenin protein level. (A)-(B) The level of active β-catenin after BIO or JW74 treatment was determined by western blotting. (C) The protein level of WNT4 and β-catenin in PGCs treated with inhibitors or si*Tet1* was measured. *Tet1*, translocation 1; PGCs, primordial germ cells. ^a–d^ Means having different letters are different (p<0.05) (n = 3).

In this study, the level of β-catenin was detected to investigate the possible mechanism underlying the activity of *Tet1*. The culture medium was added with 0.5, 1.0, and 1.5 μM BIO or 5.0, 10, and 15 μM JW74. Result revealed that only a modest increase in the level of β-catenin was observed in 0.5 μM BIO, while 1 μM BIO showed substantial increase in β-catenin level (Figure 3A) (p<0.05). By contrast, the level of β-catenin remarkably reduced at 10 μM and 15 μm JW74 treated PGCs (Figure 3B) (p<0.05). The level of β-catenin was rescued in *Tet1* knockdown group with addition of BIO, but it was still downregulated significantly compared with control (Figure 3C) (p<0.05).

### *Tet1* regulated PGC proliferation via Wnt4/β-catenin signaling pathway

**Figure 4:**
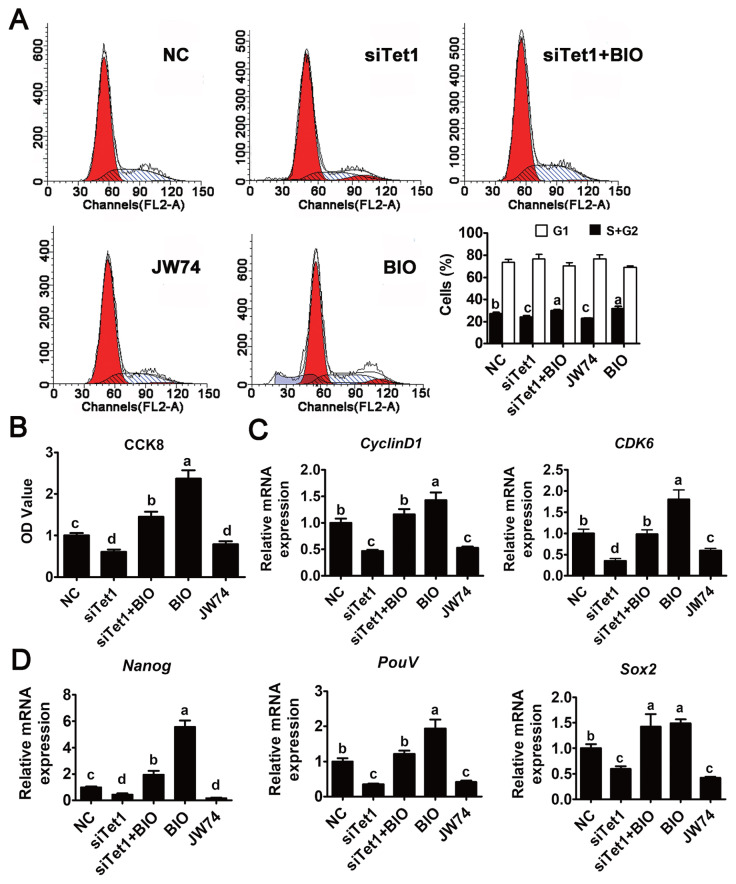
Effects of *Tet1* knockdown on PGC proliferation via Wnt4/β-catenin signaling pathway. (A) Detection of cell cycle was conducted in *Tet1* knockdown and inhibitor treated PGCs by flow cytometry. (B) Cell growth of each group was measured using the CCK8 assay. (C) The expression of cell cycle-related genes (*Cyclin D1* and *CDK*6) was tested by qRT-PCR. (D) The expression of pluripotency-related genes *PouV*, *Nanog*, and *Sox2* was detected by qRT-PCR. *Tet1*, translocation 1; PGCs, pimordial germ cells; CCK8, cell counting kit-8; *CDK6*, Cyclin-dependent kinase 6; *PouV*, POU domain class 5; *Nanong*, Nanog homeobox; *Sox2*, Sex-determining region Y-box2; qRT-PCR, quantitative real time polymerase chain reaction. ^a–d^ Means having different letters are different (p<0.05) (n = 3).

To determine whether *Tet1* exerted the proliferative effect through Wnt4/β-catenin signaling pathway, siTet1 was added along with BIO in PGC culture and the cell-cycle phase distribution was first measured. Results indicated that a notable decrease in G2/M phase was observed in cells treated with si*Tet1* and JW74 (Figure 4A) (p<0.05), demonstrating that PGC proliferation was significantly inhibited by JW74. By contrast, cell cycle distribution analysis showed the accumulation of cells in S and G2/M phase and a decrease in the proportion of cells in G1 phase after incubation with BIO (Figure 4A) (p<0.05). Cell counting showed there to be less cell proliferation in BIO and siTet1 than that in control group or group treated with BIO alone (Figure 4A) (p<0.05), indicating the proliferative effect of *Tet1* on chicken PGCs was induced via the Wnt4/β-catenin signaling pathway. Moreover, the proliferative capacity of cultured PGCs was examined by using the CCK8 assay. As shown in Figure 4B, the growth rate of the siRNA and JW74 group decreased significantly compared with the control group, while the BIO and si*Tet1* combined with BIO group increased PGC proliferation significantly (p<0.05). To further elucidate the effect of *Tet1* knockdown on PGCs, the expression of cell cycle and pluripotency related genes was examined. *Tet1* knockdown blocked the mRNA expression of *cyclin D1*/*CDK6*, and consequently delayed the entrance to G1/S phase (p<0.05) (Figure 4C). However, this inhibition was rescued by the addition of *BIO*. To further investigate the role of *Tet1* in the pluripotency of PGCs via Wnt4/β-catenin signaling pathway, the expression of pluripotency related genes *Nanog*, *Sox2*, and *PouV* was evaluated, in si*Tet1* and JW74 group, all those three genes were significantly downregulated, while they were significantly elevated in BIO and si*Tet1* combined with BIO group (Figure 4D) (p<0.05). These results, along with the dot blot results shown previously, confirmed that *Tet1* induced the accumulation of 5hmC in PGCs to promote their proliferation and knockdown of *Tet1* in PGCs led to inhibiting cell growth via Wnt4/signaling pathway.

### *Tet1* knockdown affected the status of methylation in PGCs

**Figure 5:**
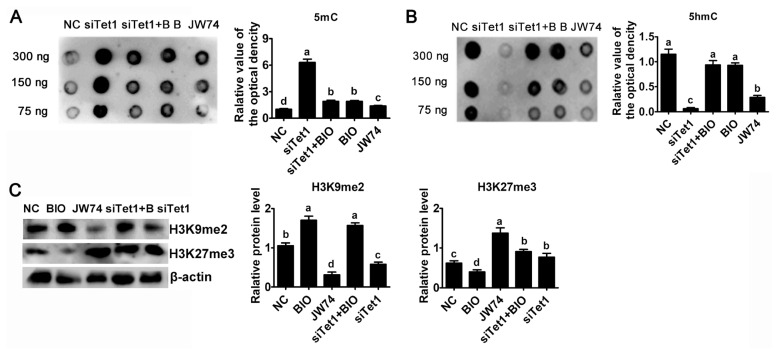
Effects of *Tet1* knockdown on DNA and histone methylation. The levels of 5mC (A) and 5hmC (B) in PGCs treated with inhibitors or si*Tet1* was measured by DNA dot blot. (C) H3K9me2 and H3K27me3 protein level was assessed using western blotting and densitometric evaluation. β-actin was used as an internal control. All the results represent the mean±standard deviation of three independent experiments (n = 3). *Tet1*, translocation 1; PGCs, primordial germ cells. ^a–d^ Means having different letters are different (p<0.05).

To investigate whether *Tet1* is required for maintaining the hypomethylated state, the abundance of 5mC and 5hmC in *Tet1* knockdown PGCs was estimated by using dot blot. Result reflected an obviously trend for increasing 5mC level in *Tet1* knockdown PGCs was observed (Figure 5A) (p<0.05). *Tet1* knockdown as well as JW74 significantly impaired the accumulation of 5hmC, whereas addition of BIO resulted in a marked increase in 5hmC level (Figure 5B) (p<0.05). After disrupting the level of 5hmC in PGCs by down-regulating *Tet1*, there was no significant change of 5hmC status in BIO and si*Tet1* combined BIO group (Figure 5B) (p<0.05). As shown in Figure 5B, treatment with siTet1 as well as JW74 prevented 5hmC accumulation in PGCs (p<0.05).

Next, whether histone demethylation plays any roles in chicken PGCs, the protein level of H3K9me2 and H3K27me3 were probed by western blot. Interestingly, the H3K27me3 global level was elevated, whereas the H3K9me2 level was greatly reduced in si*Tet1* group compared to control group, but BIO reduced H3K27me3 level and elevated H3K9me2 level (Figure 5C) (p<0.05). In addition, si*Tet1* with BIO elevated both H3K9Mme2 and H3K27me3 (Figure 5C) (p< 0.05). Moreover, we found that the level of H3K27me3 was important for chicken PGC proliferation.

## DISCUSSION

DNA methylation is an epigenetic modification that influences gene expression [24,25]. TET family comprises three members have the capacity to regulate 5mC and 5hmC levels [26]. However, the mechanism by which TET1 is involved in methylation status in PGCs and proliferation program remains unclear. In the present study, knockdown of *Tet1* exerted an obvious effect on 5hmC accumulation in PGCs, which probably reflected the diminished capacity of these cells to reprogram. As a result, the proliferation of *Tet1* knockdown PGCs was impaired.

TET1 is known to promote DNA demethylation, which influences gene transcription, especially the genes related to proliferation and differentiation in ESCs, muscle progenitor cells and cancer cells [27]. TET1 and TET2 proteins are expressed by pluripotent ESCs [11], regulated by Oct4, and have been implicated in DNA demethylation during PGC development [28,29]. Although Tet1-null female mice were recently shown to have reduced number of germ cells, they fail to properly reactivate meiotic genes [29]. Previous study observed that the loss of *Tet1* downregulated a cohort of genes involved in the proliferation of adult neural progenitor cells [27]. The reduction of *Tet1* significantly suppressed human dental pulp cells (hDPCs) growth, thus inhibited the capacity of hDPCs to differentiates into odontoblasts-like-cells and generated reparative dentin in response to exogenous stimuli or injury [27]. Recent studies of developing mouse PGCs suggested that the progressive loss of DNA methylation in cells from E8 onward occurs in discrete temporal phases in which different genic and intergenic regions are affected [30]. Several studies reported that the paradoxical phenomenon of TET1 causing both the upregulation of differentiation-related genes and downregulation of pluripotency-related genes [1,27]. In addition, many other studies also demonstrated that *Tet1* knockdown caused more genes to be upregulated than to be downregulated [9,14].

Specific gene expression mediated by β-catenin activation generally occurs in a cell-context and cell-dependent manner [15,31]. *CyclinD1* and Myc are the best-known target genes that enhance proliferation [31]. Furthermore, JW74 treatment caused the downregulation of cell cycle-related genes essential for entry in G1 phase, such as *CyclinD1* and *CDK6* [31], which is consistent with this study. In addition, depletion of *Tet1* had significant effects on PGC proliferation. Recent study demonstrated that *Tet1* exerted tumor suppressive effects in CRC cells [14]. The present study further investigated the role of *Tet1* in the proliferation of PGCs and suggested that *Tet1* affected the proliferation of chicken PGCs via Wnt4/β-catenin signaling pathway.

Despite the apparent effects on gene expression and PGC proliferation, *Tet1* depletion also exerted vital effects on DNA methylation. Global DNA demethylation is a distinguishing epigenetic feature of developing PGCs [27]. TET1 plays a crucial role in the DNA demethylation and has a profound impact on cell self-renewal [27]. Numerous studies have proven that TET1 is important in the modulation of PGC formation, embryo development due to it could control active and passive demethylation via different mechanisms [27]. Previous research revealed that *Tet1* regulated neural progenitor cell proliferation in adult mouse brain and mice lacking *Tet1* exhibited impaired hippocampal neurogenesis accompanied by poor learning and memory [32]. TET-mediated 5mC oxidation counterbalances *de novo* methylation to ensure their responsiveness to transcriptional activation driven by upstream signaling pathways and in the absence of TET, abnormal methylation led to a dysregulated Lefty-Nodal circuit [33]. In this study, significant change in 5hmC and 5mC levels was detected after *Tet1* knockdown. These results determined the loss of *Tet1* contributed to aberrant DNA methylation.

To test whether TET dioxygenase activity is required for Wnt signaling pathway and histone modification, knockdown of *Tet1* specifically abolished the enzymatic activity in chicken PGCs. A recent study showed that cell-cycle-related and expression-elevated protein in tumor (CREPT) and p15INK4b-related sequence/regulation of nuclear pre-mRNA domain-containing protein 1A (p15RS) regulated cell proliferation and the cell-cycle transition in chicken embryo fibroblast cells by mediating the transcription of Wnt/β-catenin signaling pathway downstream regulatory genes [34]. Long non-coding RNAs (lncRNAs) H19 facilitated proliferation and inhibited apoptosis through modulating Wnt/β-catenin signaling pathway [35]. In this study, it was found that si*Tet1* inactivated β-catenin in PGCs cultured *in vitro*. The expression level of H3K9me2 and H3K27me3 changed because of *Tet1* knockdown, and a lower level of H3K9me2 and a higher level of H3K27me3 than that of control were observed.

In mice, epigenetic reprogramming of the germ cells specified in the late epiblast occurs when they migrate and settle in the gonads [4,5]. Loss of dimethylation of H3K9 and DNA methylation, concomitant with an enhancement of H3K27 and H3K4 trimethylation and histone acetylation, occurs at E8.5. In avian species, PGCs had a unique chromatin conformation, and the global level of H3K9me3 was higher in PGCs than in chicken ESCs [20]. On the contrary, the level of H3K27me3 was lower in PGCs than in chicken ESCs, and, most importantly, the nuclear distribution of this PTM was strikingly different, with H3K27me3 only detectable at one large spot similar to ESC chromocentres [20]. H3K27me3-based repression may partially replace H3K9me3-based repression, given that differentiation induces a decrease of H3K9me3 global level. PGCs follow a different epigenetic path in which H3K9me3 prevails over H3K27me3.

## CONCLUSION

This study showed that *Tet1* played distinct roles in proliferation and pluripotent reprogramming in chicken PGCs. Loss of 5hmC, accumulation of 5mC and reduction of pluripotency-associated genes, as well as drastic decrease of proliferation, were observed with knockdown of *Tet1*, which indicated that Tet1 was related with the methylation state to regulate the identification and expansion of PGCs in chickens. Collectively, these findings revealed a fundamental mechanism that *Tet1* enhanced the proliferation of chicken PGCs via the activation of Wnt4/β-catenin signaling pathway which is achieved through regulating demethylation status.

## Figures and Tables

**Table 1:** The primer sequences for quantitative real time polymerase chain reaction

Gene	Accession No.	Primer sequences (5′ to 3′)	Length (bp)
*β* *-actin*	NM_205518	F: ACGTCGCACTGGATTTCGAG R: TGTCAGCAATGCCAGGGTAC	282
*Tet1*	XM_015278732	F: CAGGAGCAGTGTCACCAGAA R: TGCAATTGTTGGAGCTTGAG	166
*Sox2*	NM_205188	F: AGAGAAAAGGGAAAAAGGA R:TTTCCTAGGGAGGGGTATGAA	171
*PouV*	NM_001309372	F: GTTGTCCGGGTCTGGTTCT R: TGGAAAGGTGGCATGTAGAC	187
*Nanog*	NM_001146142	F: CAGCAGACCTCTCCTTGACC R: TTCCTTGTCCCACTCTCACC	216
*CDK6*	NM_001007892	F:CCGACCAACAGTATGAGTGCG R: AAAATCCAGTCCCCGAAACA	231
*CyclinD1*	NM_205381	F: GTTGTCCGGGTCTGGTTCT-3′ R: TGGAAAGGTGGCATGTAGAC	221

*Tet1*, translocation 1; PGCs, pimordial germ cells; *Sox2*, Sex-determining region Y-box 2; *PouV*, POU domain class 5; *Nanong*, Nanog homeobox; *CDK6*, Cyclin-dependent kinase 6.
